# PlGF/FLT-1 deficiency leads to reduced STAT3-C/EBPβ signaling and aberrant polarization in decidual macrophages during early spontaneous abortion

**DOI:** 10.3389/fimmu.2023.1061949

**Published:** 2023-03-15

**Authors:** Ruiqi Chang, Jingcong Dai, Li Wang, Hao Liu, Huanhuan Jiang, Xiaoyu Liu, Linying Jiang, Fan He, Lina Hu

**Affiliations:** ^1^ The Center for Reproductive Medicine, Department of Obstetrics and Gynaecology, The Second Affiliated Hospital of Chongqing Medical University, Chongqing, China; ^2^ Joint International Research Lab for Reproduction and Development, Ministry of Education, Chongqing, China; ^3^ Reproduction and Stem Cell Therapy Research Center of Chongqing, Chongqing Medical University, Chongqing, China; ^4^ Department of Obstetrics and Gynaecology, The Second Affiliated Hospital of Chongqing Medical University, Chongqing, China

**Keywords:** placental growth factor, Fms-like-tyrosine-kinase receptor 1, decidual stromal cells, decidual macrophage polarization, spontaneous abortion, early pregnancy

## Abstract

**Introduction:**

Dysregulated macrophage polarization (excessive M1-like or limited M2-like macrophages) in the early decidua contributes to allogeneic fetal rejection and thus early spontaneous abortion. However, the modulators of M1/M2 balance at the early maternal-fetal interface remain mostly unknown.

**Methods:**

First-trimester decidual tissues were collected from normal pregnant women undergoing elective pregnancy terminations and patients with spontaneous abortion. We measured the expression of placental growth factor (PlGF) and Fms-like-tyrosine-kinase receptor 1 (FLT-1), and characterized the profiles of macrophages in decidua. Notably, we investigated the effect of recombinant human PlGF (rhPlGF) on decidual macrophages (dMφs) from normal pregnancy and revealed the underlying mechanisms both *in vitro* and *in vivo*.

**Results:**

The downregulated expression of PlGF/ FLT-1 may result in spontaneous abortion by inducing the M1-like deviation of macrophages in human early decidua. Moreover, the CBA/J×DBA/2 abortion-prone mice displayed a lower FLT-1 expression in uterine macrophages than did CBA/J×BALB/c control pregnant mice. In *in vitro* models, rhPlGF treatment was found to drive the M2-like polarization of dMφs via the STAT3/CEBPB signaling pathway. These findings were further supported by a higher embryo resorption rate and uterine macrophage dysfunction in Pgf knockout mice, in addition to the reduced STAT3 transcription and C/EBPβ expression in uterine macrophages.

**Discussion:**

PlGF plays a key role in early pregnancy maintenance by skewing dMφs toward an M2-like phenotype via the FLT-1-STAT3-C/EBPβ signaling pathway. Excitingly, our results highlight a rationale that PlGF is a promising target to prevent early spontaneous abortion.

## Introduction

During early pregnancy, decidual stromal cells (DSCs) provide immune privilege by secreting a series of chemokines, cytokines, and growth factors, and thus participate in embryo implantation and placental development (1). On the one hand, DSCs ensure that the maternal-fetal interface is abundantly populated by immune-suppressive cells, including specialized decidual natural killer (dNK) cells, decidual macrophages (dMφs), T helper 2 (Th2) cells, and regulatory T cells (Tregs) (2–8). On the other hand, DSCs limit the accumulation and function of Th1 and cytotoxic T cells in the decidua by silencing *Cxcl9*, *Cxcl10*, *Cxcl11*, and *Ccl5* (9, 10). Aberrant DSCs may induce a prolonged and disordered proinflammatory response, which may lead to adverse pregnancy outcomes such as early spontaneous abortion (1, 11, 12).

As the second largest population in decidual immune cells (DICs), dMφs participate in several physiological processes which are essential for a successful pregnancy, including maternal immune tolerance (9). With its high plasticity and heterogeneity, dMφs undergo dynamic and highly regulated changes throughout pregnancy (13). It has been reported that dMφs exhibit a mixed M1/M2 profile with M2-predominance since trophoblasts attach to maternal decidua, and this pattern runs through the first trimester (14). However, the modulators of M1/M2 balance in the early decidua remain mostly unknown.

Placental growth factor (PlGF), a member of the vascular endothelial growth factor (VEGF) family, is a pivotal regulator for the onset and maintenance of early pregnancy and secreted by DSCs, trophoblast cells, and dNK cells (15–17). Up to the present, Fms-like-tyrosine-kinase receptor 1 (FLT-1) is the only known signaling receptor for PlGF in humans and expresses on endothelial cells, osteoclasts, smooth muscle cells, fibroblasts, angiogenesis-competent myeloid progenitors, tumour cells, T cells, and monocyte/macrophage lineage cells (18–20). PlGF/FLT-1 is best known for its involvement in placental angiogenesis and maternal spiral arteries remodeling *via* regulating physiological activities of endothelial cells during early pregnancy (18, 21, 22). Notably, FLT-1 also expresses on macrophages, and PlGF plays a modulatory role in the differentiation and maturation of human dendritic cells (23). However, the action of PlGF in macrophage polarization is rarely investigated. In this study, we focus on the effect of PlGF on the polarization of dMφs and reveal the potential relationship between decreased PlGF secretion and early spontaneous abortion.

## Materials and methods

### Human samples

Decidual tissues were collected from women with normal pregnancy (undergoing elective terminations, n=85) and spontaneous abortion (n=31) at 6-10 weeks of gestation. All subjects were confirmed by ultrasound. We excluded the spontaneous abortion with infection, chromosomal abnormality, anatomic defects, glucose metabolism disorders, thyroid dysfunction, or potential autoimmune diseases (where the anti-phospholipid antibodies or antinuclear antibodies were positive). The demographic and clinical characteristics of women with normal pregnancy and spontaneous abortion are summarized in **Supplemental Table 1**. All decidual samples were immediately kept in ice-cold sterile DMEM/F12 medium (HyClone) and transported to the laboratory for further processing within 30 min after the operation.

### Mice

Female CBA/J mice (RRID : IMSR_JAX:000656), male DBA/2 mice (RRID : IMSR_JAX:000671), male BALB/cJ mice (RRID : IMSR_JAX:000651) and wildtype C57BL/6NJ mice (RRID : IMSR_JAX:005304) were purchased from Jackson Laboratory. After adaptive feeding, female CBA/J mice were mated to male BALB/c or DBA/2 mice to establish the models of normal pregnancy (CBA/J × BALB/c, n=5) or spontaneous abortion (CBA/J × DBA/2, n=6). The fertilized eggs of C57BL/6 mice were microinjected with Cas 9/guide RNA complexes targeting at exons 2, 3, and 4 of the murine *Pgf*, incubated in a 5% CO_2_ incubator at 37°C until the two‐cell stage, and then transferred to foster mothers. Then the F1 heterozygotes were generated by the backcrossing of genome‐modified F0 generation mice with wild‐type mice, and confirmed by qRT-PCR. To avoid the confounding effect of embryonic genotype, the heterozygotes were mated with wild-type mice to generate pregnant mice in the experimental group (*Pgf^-^* ^/+^ females × wild-type males, n=24) and control group (wild-type females × *Pgf^-^* ^/+^ males, n=26).

### Primary cell isolation, purification, and culture

As previously described, human DICs and DSCs were isolated from decidual tissues, and murine uterine immune cells were isolated from uterine tissues at embryonic day 8.5 (24). Human dMφs were then purified from DICs using Anti-CD14 MicroBead Kit (Miltenyi, Cat #130-0490601). The purity of CD45^+^ DICs (above 98%), Vimentin^+^CD45^-^ DSCs (above 98%), CD45^+^CD14^+^ dMφs (above 95%), and CD45^+^ murine uterine immune cells (above 98%) were validated by flow cytometry (FCM) analysis. The primary isolated cells were cultured in RPMI 1640 (HyClone) or DMEM/F12 (HyClone) medium containing 10% fetal bovine serum (FBS, Gibco, Australia) in 5% CO_2_ at 37°C.

### Cell line

The human epithelial cell line 293-T was purchased from American Type Culture Collection (ATCC, CRL-3216) and cultured in DMEM (HyClone) medium containing 10% FBS (Gibco, Australia) in 5% CO_2_ at 37°C.

### Immunohistochemistry

Human decidual tissues were labeled with rabbit anti-PlGF (Abcam) for immunohistochemical staining as previously described (24). Specifically, the formalin-fixed decidual tissues were dehydrated in ethanol, embedded with paraffin, and cut into slices. After the deparaffinization and rehydration, the sections were pretreated with sodium citrate buffer (0.01M, pH=6.0) for antigen retrieval, followed by blocking the endogenous peroxidase activity with 3% H_2_O_2_ and 5% bovine serum albumin. Then, the sections were incubated with rabbit anti-PlGF antibodies (1:100, Abcam), or rabbit IgG overnight at 4°C. After washing with PBS, the sections were incubated with secondary antibody for 30min at room temperature, labeled with 3,3’-diaminobenzidine, and counterstained with hematoxylin.

### Quantitative real-time PCR

Total RNA was extracted using the TRIzol reagent (Invitrogen) and then reverse-transcribed into cDNA using the PrimeScript RT Master Mix (Takara). The qRT-PCR analysis was performed using the TB Green Premix EX Taq II (Takara) and the CFX96 Real-Time system (Bio-Rad). The primer sequences are shown in **Supplemental Table 2**. Each sample was analyzed in triplicate. Relative mRNA levels were normalized to *GAPDH*.

### Enzyme-linked immunosorbent assay

After 48-hour culture of freshly isolated DSCs, the concentration of PlGF was measured by Human PlGF Quantikine ELISA kit (R&D Systems) according to the manufacturer’s instructions.

### FCM

For FCM analyses, cells were incubated with monoclonal antibodies (**Supplemental Table 3**) according to the manufacturers’ instructions. Intracellular and intranuclear staining was performed after the fixation and permeabilization with Fixation/Permeabilization Solution Kit (BD Pharmingen) and Transcription Factor Buffer Set (BD Pharmingen) respectively. FCM was performed using Beckman-Coulter CyAN ADP Analyzer (Beckman-Coulter) and analyzed by FlowJo software (version 10.0, TreeStar).

### Dual-luciferase reporter assays

With the help of the JAS-PAR database, we predicted the binding site of the *CEBPB* promoter to STAT3 and introduced mutations in the predicted site. 293-T cells were seeded in 24-well plates (2×10^5^ cells per well) and cotransfected with STAT3-overexpression or vector plasmids, wild-type or mutant *CEBPB* promoter-reporter plasmids, and TK promoter-Renilla plasmids (GeneChem Co.) using Lipofectamine 2000 (Invitrogen). After 48 hours of transfection, dual-luciferase reporter assays were performed with the dual-luciferase reporter assay kit (Promega) according to the manufacturer’s instructions. The primer sequences are shown in **Supplemental Table 4**.

### Statistical analysis

Continuous variables are shown as mean ± SEM. The statistical comparison between two groups was performed using t-test when they are normally distributed or Wilcoxon test if not. The correlation analysis of two continuous variables was performed by the Spearman correlation analysis. All analyses were conducted with IBM SPSS statistics 25.0. A two-tailed *P*-value < 0.05 was considered statistically significant.

### Ethics

All procedures of this study were approved by All participants provided informed consent as part of the protocols approved by the Research Ethics Committee of the Second Affiliated Hospital, Chongqing Medical University (Chongqing, China, #2020-150). And all animal procedures were approved by the Animal Care and Use Committee of Chongqing Medical University.

## Results

### The reduction of PlGF/FLT-1 expression in the decidua from early spontaneous abortion

**Figure 1:**
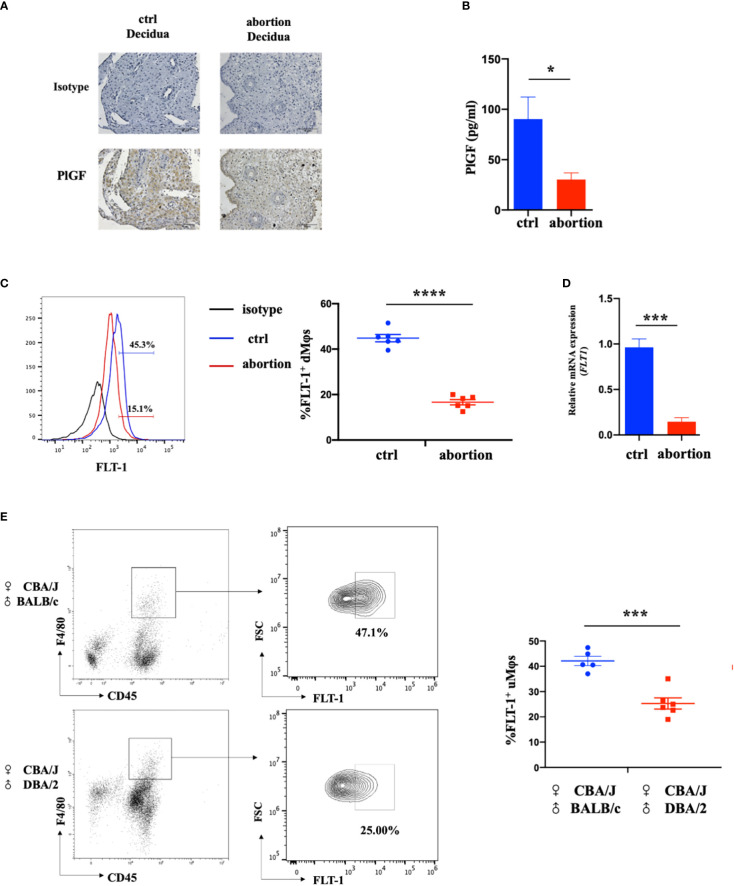
The expression of DSC-derived PlGF and its receptor FLT-1 in the early decidua. **(A)** Immunohistochemistry analysis of PlGF expression in the decidua from normal pregnancy (ctrl) and spontaneous abortion (n=3). Original magnification: ×200. **(B)** The level of PlGF secreted by DSCs (1×10^5^ cells per well) from normal pregnancy (ctrl) and spontaneous abortion (n=8) by ELISA at 48 hours after culture. **(C)** Flow cytometry (FCM) analysis of FLT-1 expression in decidual macrophages (dMφs) from normal pregnancy (ctrl) (n=6) and spontaneous abortion (n=6). **(D)** The qRT-PCR analysis of *FLT-1* expression in dMφs from normal pregnancy (ctrl, n=4) and spontaneous abortion (n=4). **(E)** FCM analysis of FLT-1 expression on uterine macrophages (uMφs) from CBA/J×BALB/c control pregnant mice (n=5) and CBA/J×DBA/2 abortion-prone mice (n=6) in the first trimester. Data were analyzed by Student’s t-test and shown as mean ± SEM. *P < 0.05, ***P < 0.001, and ****P < 0.0001. Ctrl, control.

To investigate the possible role of DSC-derived PlGF in the maintenance of early pregnancy, we firstly analyzed the expression of *PGF* in DSCs from normal pregnancy and spontaneous abortion during early pregnancy. In comparison with normal pregnancy, DSCs from spontaneous abortion showed a weaker staining of PlGF in the cytoplasm (**Figure 1A**). Furthermore, a markedly lower PlGF secretion in DSCs from spontaneous abortion was demonstrated by ELISA (**Figures 1B**). These results suggest that the decreased level of DSC-derived PlGF may be related to spontaneous abortion.

FLT-1 is the only known signaling receptor for PlGF in humans and expresses on macrophages (18). We then investigated the expression of *FLT-1* on dMφs in the first trimester. Interestingly, with the help of FCM, FLT-1^+^ macrophages were found in the early decidua and a prominent reduction of FLT-1^+^ dMφs was observed in patients with spontaneous abortion when compared with normal pregnancy (16.65 ± 1.06% vs. 44.83 ± 1.47%) (**Figure 1C**). The mRNA level of *FLT1* in dMφs from patients with spontaneous abortion was also lower than that of normal pregnancy (**Figure 1D**). Consistent with this, CBA/J×DBA/2 abortion-prone mice displayed fewer FLT-1^+^ uterine macrophages than did CBA/J×BALB/c control pregnant mice (25.63 ± 2.39% vs. 42.10 ± 1.63%) (**Figure 1E**). These findings indicate that the reduction of PlGF/FLT-1 expression in decidua may result in early spontaneous abortion.

### The decreased PlGF/FLT-1 expression contributed to the imbalance of M1/M2 paradigm at the maternal-fetal interface in early spontaneous abortion

**Figure 2:**
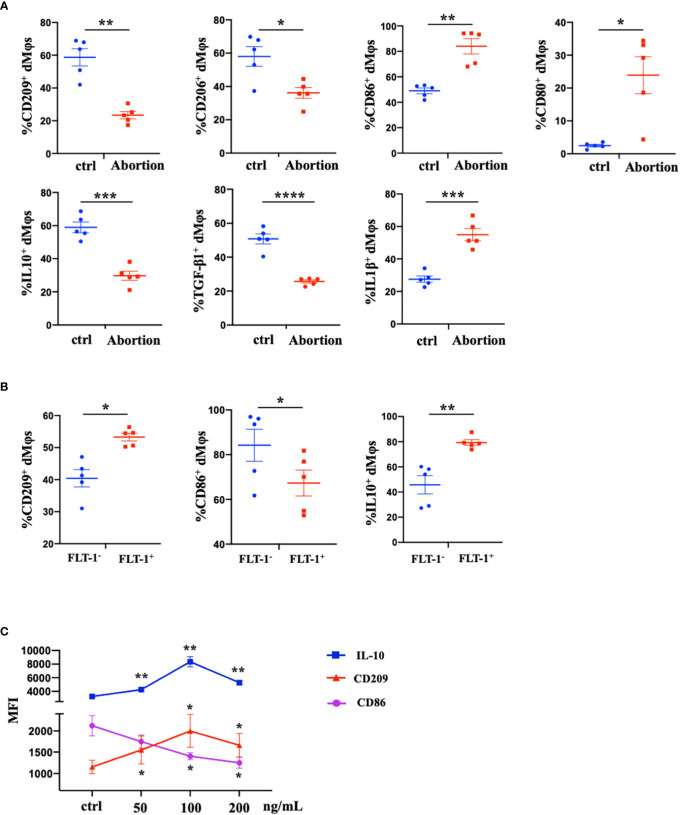
The decreased PlGF/FLT-1 expression contributed to the imbalance of M1/M2 paradigm at the maternal-fetal interface in early spontaneous abortion. **(A)** Flow cytometry (FCM) analysis of immune profiles (the expression levels of CD209, CD206, CD86, CD80, IL-10, TGF-β1, and IL-1β) in decidual macrophages (dMφs) from normal pregnancy (n=5) and spontaneous abortion (n=5). **(B)** The expression levels of CD209, CD86, and IL-10 in FLT-1^−^ (n=5) and FLT-1^+^ dMφs (n = 5) by FCM analysis. **(C)** The MFI levels of CD86, CD209, and IL-10 in dMφs by FCM analysis after 48 hour treatment with recombinant human PlGF (rhPlGF) at gradient concentrations (5 independent experiments). Data were analyzed by Student’s t-test, Wilcoxon rank-sum test, paired t-test, or Wilcoxon matched-pair signed-ranks test, and shown as mean ± SEM. *P < 0.05, **P < 0.01, ***P < 0.001, and ****P < 0.0001. ctrl, control; MFI, mean fluorescence intensity.

We further characterized the profiles of macrophages in the early decidua from normal pregnancy and spontaneous abortion. A higher M1/M2 ratio of dMφs was demonstrated in spontaneous abortion, which manifested a higher expression of M1-associated markers (CD86, CD80, and IL-1β) and lower expression of M2-associated markers (CD209, CD206, IL-10, and TGF-β1 (**Figure 2A**). As previously described, a significant reduction of FLT-1^+^ dMφs was observed in early spontaneous abortion. Interestingly, compared to the FLT-1^-^ subtype, FLT-1^+^ dMφs in the early decidua exhibited an M2-like profile, with a higher expression of M2-associated markers (CD209 and IL-10) and a lower expression of M1-associated markers (CD86) (**Figure 2B**). Thus, we hypothesized that the reduction in FLT-1^+^ dMφs might be associated with the imbalance of M1/M2 in spontaneous abortion. To confirm this, we assessed the expression levels of M1- and M2-associated markers in primary dMφs treated with rhPlGF for 48 hours. As shown in **Figure 2C**, the rhPlGF-polarized (100 ng/mL) dMφs exhibited an M2-like profile, with an increased expression of CD209 and IL-10, as well as a decreased expression of CD86. Also, the enhancing effect on CD209 expression was found in primary dMφs treated with rhPlGF at the concentration of 50 ng/mL, and the suppressing effect on CD86 expression was found at the concentrations of 50 ng/mL and 200 ng/mL (**Figure 2C**). However, the expression of other M1- and M2-associated markers (CD206, CD80, TGF-β1, and IL-1β) remained unaltered after rhPlGF treatment (data not shown). These data suggest that the decreased PlGF/FLT-1 expression may participate in early spontaneous abortion by inducing the M1-like deviation of dMφs.

### PlGF promoted the M2-like polarization of dMφs *via* the STAT3-C/EBPβ signaling pathway

**Figure 3:**
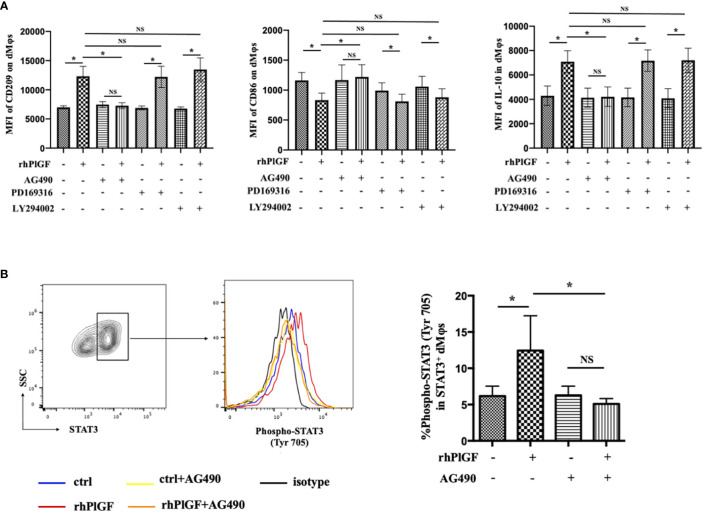
PlGF drived the M2-like polarization by promoting STAT3 phosphorylation at Tyr 705 in macrophages from early decidua. **(A)** The MFI levels of CD209, CD86, and IL-10 in decidual macrophages (dMφs) by flow cytometry (FCM) analysis after 48 hour treatment with recombinant human PlGF (rhPlGF, 100 ng/mL) following 1 hour pretreatment with AG490 (10 μM), LY294002 (10 μM) or PD169316 (10 μM) (5 independent experiments). **(B)** The phosphorylation level of STAT3 at tyrosine 705 (Tyr 705) in dMφs by FCM analysis after 48 hour treatment with rhPlGF (100 ng/mL) following 1 hour pretreatment with AG490 (10 μM) (4 independent experiments). Data were analyzed by paired t-test or Wilcoxon matched-pair signed-ranks test, and shown as mean ± SEM. *P < 0.05, and *NS* no statistically difference. MFI, mean fluorescence intensity.

Previous studies revealed that the stimulation of FLT-1 by PlGF leads to the activation of downstream signaling molecules, including STAT3, PI3 kinase (PI3K), and p38 MAP kinase (p38 MAPK) (25, 26). To identify the downstream pathway through which PlGF drives the M2-like polarization of macrophages in the early decidua, we analyzed the profiles of rhPlGF-polarized dMφs following the pretreatment with selective inhibitors of STAT3 (AG490), PI3K (LY294002) or p38 MAPK (PD169316). Notably, the expression changes of CD209, IL-10, and CD86 caused by rhPlGF treatment (100 ng/mL) were only almost abrogated by AG490, a STAT3 inhibitor (**Figure 3A**). As is known, STAT3 forms homodimers by tyrosine phosphorylation at position 705, which is essential for the transcriptional function of STAT3 (27). Moreover, the transcriptional activity of STAT3 can also be enhanced by serine phosphorylation at position 727 (28). We further assessed the phosphorylation status of STAT3 at tyrosine 705 (Tyr 705) and serine 727 (Ser727) in primary dMφs treated with rhPlGF. Compared with the control, the rhPlGF-polarized (100 ng/mL) dMφs showed a significantly higher tyrosine phosphorylation level of STAT3 at position 705 (**Figure 3B**), while a similar serine phosphorylation level at position 727 (data not shown). In addition, the rhPlGF-promoted STAT3 phosphorylation was profoundly inhibited by AG490 (**Figure 3B**). These results suggested that PlGF may drive the M2-like polarization of dMφs by up-regulating the phosphorylation level of STAT3 at Tyr 705 and enhancing the transcriptional activity of STAT3.

**Figure 4:**
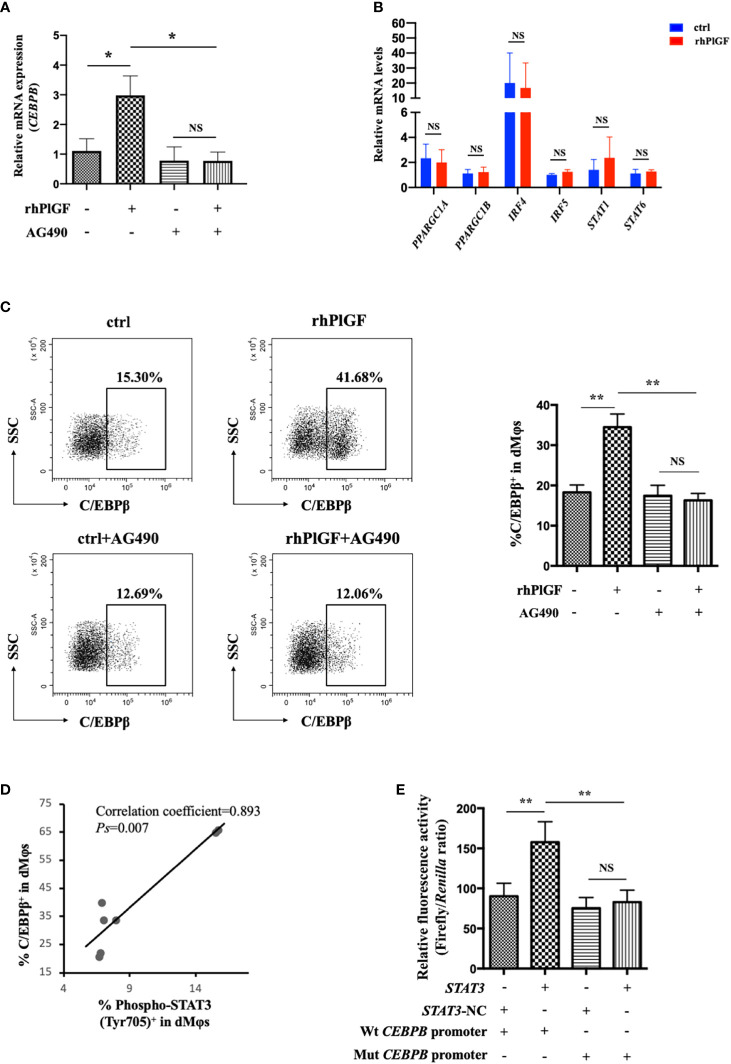
PlGF promoted the M2-like polarization *via* the STAT3-C/EBPβ signaling pathway. **(A)** The qRT-PCR analysis of *CEBPB* expression in decidual macrophages (dMφs) after 48 hour treatment with recombinant human PlGF (rhPlGF, 100 ng/mL) following 1 hour pretreatment with AG490 (10 μM) (4 independent experiments). **(B)** Relative mRNA levels of *PPARGC1A, PPARGC1B, IRF4, IRF5, STAT1*, *and STAT6* in dMφs after 48 hour treatment with rhPlGF (100 ng/mL) (3 independent experiments). **(C)** The expression level of C/EBPβ in dMφs by FCM analysis after 48 hour treatment with rhPlGF (100 ng/mL) following 1 hour pretreatment with AG490 (10 μM) (9 independent experiments). **(D)** The Pearson correlation analysis for the levels of C/EBPβ expression and STAT3 phosphorylation at Tyr 705 in rhPlGF-polarized (100 ng/mL) dMφs (n=7). **(E)** STAT3-overexpression or vector plasmids, wild-type or mutant *CEBPB* promoter-reporter plasmids, and TK promoter-Renilla plasmids were co-transfected in the 293T cells for 48 hours. The luciferase activity was measured and normalized to Renilla luciferase (9 independent experiments). Data were analyzed by paired t-test or Wilcoxon matched-pair signed-ranks test, and shown as mean ± SEM. *P < 0.05, **P < 0.01, and *NS* no statistically difference. ctrl, control; Mut, mutant; NC, negative control; Wt, wild-type.

To investigate the mechanism underlying PlGF-mediated dMφ polarization, we explored whether rhPlGF regulated the expression of key transcription factors involved in the M2-like polarization of macrophages. The mRNA level of *CEBPB* remarkably increased in rhPlGF-treated (100 ng/mL) dMφs compared to the control (**Figure 4A**). However, the transcription level of other transcription factors remained unaltered, including *PPARGC1A, PPARGC1B, IRF4, IRF5, STAT1*, and *STAT6* (**Figure 4B**). Then we observed that the rhPlGF treatment (100 ng/mL) also elevated the C/EBPβ level in dMφs (**Figure 4C**). Furthermore, a positive correlation was found between the expression level of C/EBPβ and the phosphorylation level of STAT3 at Tyr 705 in rhPlGF-treated (100 ng/mL) dMφs (**Figure 4D**; **Table 1**). It was noteworthy that the promoting effect of rhPlGF on the expression of *CEBPB* at both mRNA and protein levels was abrogated by AG490 pretreatment (**Figures 4A, C**). Finally, the dual-luciferase reporter assay revealed an increased luciferase activity with the co-transfection of STAT3 overexpression and wild-type *CEBPB* promoter-reporter plasmids, which was completely blocked by the mutations of the *CEBPB* promoter in its predicted binding site to STAT3 (**Figure 4E**). These data indicated that rhPlGF promotes the M2-like polarization of dMφs *via* the STAT3-C/EBPβ signaling pathway.

**Table 1:** The data for correlation analysis in **Figure 4D**.

Factors	1	2	3	4	5	6	7
% Phospho-STAT3 (Tyr 705)^+^ in dMφs	6.85	7.04	7.95	6.68	6.78	15.68	15.49
% C/EBPβ^+^ in dMφs	39.89	33.74	33.85	20.80	22.20	65.80	65.04

dMφs, decidual macrophages.

### The maternal PlGF deficiency may lead to uterine macrophage dysfunction and fetal loss in *Pgf* knockout mice

**Figure 5:**
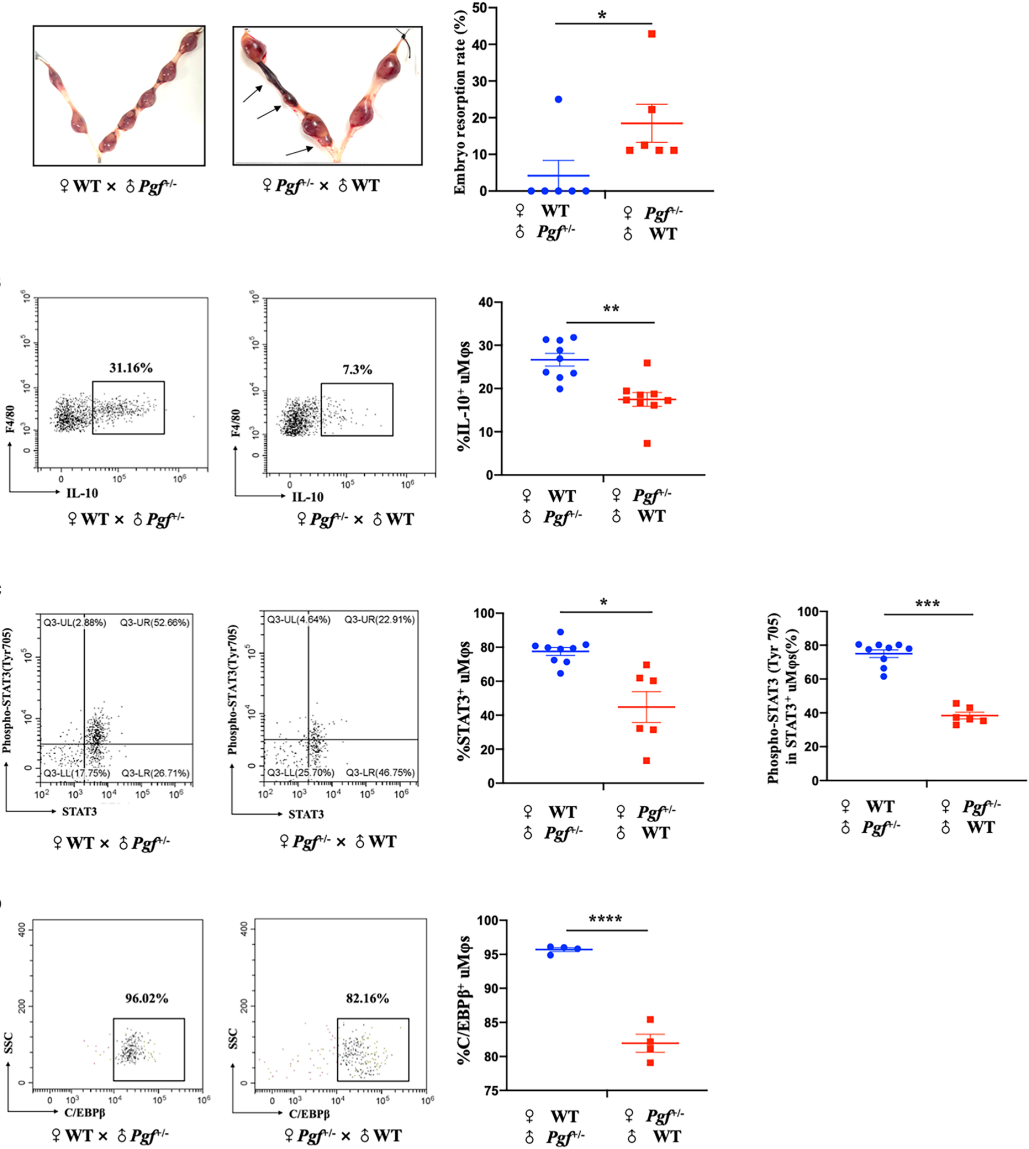
The maternal PlGF deficiency leads to uterine macrophage dysfunction and fetal loss in *Pgf* knockout mice. **(A)** The embryo resorption rate in the control group (wild-type females × *Pgf^-^* ^/+^ males, n=6) and experimental group (*Pgf^-^* ^/+^ females × wild-type males, n=6) at embryonic day 13.5. **(B)** Flow cytometry (FCM) analysis of IL-10 expression on uterine macrophagesa in the control group (n=9) and experimental group (n=9) at embryonic day 8.5. **(C)** The expression level of STAT3 and the phosphorylation level of STAT3 at tyrosine 705 (Tyr 705) in uterine macrophages in the control group (n=9) and experimental group (n=6) by FCM analysis at embryonic day 8.5. **(D)** FCM analysis of C/EBPβ expression on uterine macrophages in the control group (n=4) and experimental group (n=4) at embryonic day 8.5. Data were analyzed by Student’s t-test or Wilcoxon rank-sum test, and shown as mean ± SEM. *P < 0.05, **P < 0.01, ***P < 0.001, and ****P < 0.0001. WT, wild-type.

To validate the role of PlGF in the M2-like polarization of dMφs and the maintenance of early pregnancy *in vivo*, we constructed *Pgf* knockout mice. Unfortunately, we didn’t obtain *Pgf^-^* ^/-^ mice by mating heterozygotes, which was proved by the genotype detection. The genotypes of wild type (7/17), heterozygote (8/17) and homozygote (2/17) were detected at embryonic day 3.5, while only wild type (6/19) and heterozygote (13/19) at embryonic day 9.5. These results suggested the homozygous *Pgf* defect conferred embryo lethality. Compared to the control, the *Pgf* knockout pregnant mice showed a prominently higher embryo resorption rate at embryonic day 13.5 (**Figure 5A**), as well as a lower IL-10 production (**Figure 5B**), a lower STAT3 phosphorylation at Tyr 705 (**Figure 5C**), and a lower C/EBPβ expression (**Figure 5D**) in uterine macrophages at embryonic day 8.5. These data proved that the maternal PlGF deficiency may lead to the dysfunction of murine uterine macrophages and increase the risk of fetal loss. These findings further supported that PlGF promotes the M2-like polarization of dMφs *via* the STAT3-C/EBPβ signaling pathway during early pregnancy.

## Discussion

At the maternal-fetal interface, dMφs play an essential role in embryo implantation, maternal immune tolerance, vascular remodeling, fetal growth, and parturition initiation (9). All these functions rely on the ability of dMφs to differentiate into the classically activated M1-like phenotype or the alternatively activated M2-like phenotype in response to stimuli (29). Like Th1/Th2 cells, M1-like macrophages display pro-inflammatory functions, while M2-like macrophages have anti-inflammatory capacities (30). The polarization states of dMφs change dynamically throughout the whole period of pregnancy. At the very beginning, a mixed M1/M2 profile with M1-predominance is required for trophoblast invasion and embryo implantation (14). Since trophoblasts attaching to the maternal decidua, dMφs switch to an M2-predominance to sustain maternal immune tolerance and promote uterine vascular remodeling, then continue shifting toward the M2-like phenotype to support fetal growth after the completion of placentation, and again skew toward the M1-predominance to initiate parturition in the third trimester (13, 14, 31). Dysregulated macrophage polarization (excessive M1-like or limited M2-like macrophages) in the early decidua has been reported to be associated with allogeneic fetal rejection and finally lead to adverse pregnancy outcomes such as early spontaneous abortion (32–35). In line with previous findings, our study also revealed an inappropriate polarization pattern of dMφs in early spontaneous abortion, which was reflected by a lower expression of M2-associated markers (CD209, CD206, IL-10, and TGF-β1) and a higher expression of M1-associated markers (CD86, CD80, and IL-1β). Most importantly, we discovered that the level of PlGF secreted by DSCs and the level of FLT-1 (the only known signaling receptor for PlGF) in dMφs were significantly lower in spontaneous abortion than those in normal pregnancy. These findings indicate that the reduction of PlGF/FLT-1 in the early decidua may be associated with the dysregulated polarization of dMφs and spontaneous abortion in the first trimester. Moreover, a higher embryo resorption rate in *Pgf* knockout mice suggested that PlGF deficiency is a cause, rather than the outcome of spontaneous abortion.

As the finely regulated macrophage polarization is essential for the maintenance of early pregnancy, a growing body of studies have tried to investigate the modulators of macrophage polarization in the early decidua, including soluble HLA G5, M-CSF, Tim3, RANKL, and PD-1/PD-L1 axis (5, 33, 35–37). Recently, PlGF, a VEGF family member which consists of a 69-kD α-chain and a 34-kD β-chain linked by two intermolecular disulfide bonds, has been reported to participate in the onset and maintenance of early pregnancy (17, 18). Besides its best known function in angiogenic events, PlGF also displays the immunosuppressive capacity by skewing T cell toward the Th2 phenotype (21–23). Here, we revealed that PlGF is a potential modulator that drives the M2-like polarization of macrophages in the early decidua. We observed that PlGF promoted the expressions of an M2-associated marker CD209 and an anti-inflammatory cytokine IL-10, and suppressed the expression of an M1-associated marker CD86. On the one hand, IL-10 is one of the major anti-inflammatory cytokines generated by M2-like macrophages and protects the fetus from maternal immune attack (13). IL-10 limits inflammatory responses at the maternal-fetal interface by suppressing the production of pro-inflammatory molecules, including LPS‐induced TNF‐α, MMPs, IL‐6, and IL-8 (38–41). The *Il-10* knockout mice challenged by low-dose LPS suffered from fetal loss at a 10-fold increased risk and the exogenous addition of IL-10 alleviated the increased susceptibility (42). On the other hand, IL-10 is engaged in vascular remodeling and placentation by inducing the expression of aquaporin-1 and VEGF-C in human trophoblasts and down-regulating the Notch-dll4 axis during pregnancy (43). The absence of IL-10 gives rise to abnormal placental morphogenesis, including an enlarged placental labyrinth and pathological architecture of blood sinuses (44). Strikingly, IL-10 acts as a direct inducer of the M2-like polarization, as well as an indirect regulator by increasing the expression of IL-4R on macrophage surface and enhancing macrophage sensitivity to being directed to an M2-like phenotype, which in turn promotes the production of IL-10 (45, 46). This positive feedback between IL-10 and M2-like macrophages may greatly benefit the establishment of maternal immune tolerance and vascular remodeling in the early decidua.

Subsequently, we identified the downstream pathway through which PlGF drives the M2-like polarization of dMφs. Both the *in vitro* and *in vivo* data showed that PlGF up-regulates the tyrosine phosphorylation level of STAT3 at position 705 in macrophages at the early maternal-fetal interface. In addition, the selective inhibition of STAT3 phosphorylation leads to a complete abrogation of the promoting effect of PlGF on the M2-like polarization. STAT3 has a C-terminal tyrosine residue at position 705 and a conserved Src homology 2 (SH2) domain (47). In response to cytokine stimulation, the protein tyrosine kinase receptor signaling or intracellular protein tyrosine kinase activates, then STAT3 becomes phosphorylated at Tyr 705, followed by reciprocal interactions of SH2 domain-phosphotyrosine and the formation of homodimers (48). The homodimerization of STAT3, a critical transcriptional regulator involved in immune activities, is essential for its transcriptional function (47, 49, 50). Accordingly, we demonstrated that rhPlGF increases the transcriptional activity of STAT3 by up-regulating the tyrosine phosphorylation level of STAT3 at position 705 and thus promotes the M2-like polarization of dMφs. In bone marrow granulocytic progenitor cells, STAT3 has been reported to stimulate the expression of C/EBPβ, which was triggered by G-CSF (51). However, previous studies did not find out the direct interference of STAT3 with a wide array of pro-inflammatory gene promoters (27). To our knowledge, we revealed that STAT3 promotes the transcription of *CEBPB* by binding to its promoter. As a crucial transcriptional regulator of the M2-like polarization, C/EBPβ boosts the expression of arginase-1 and M2-associated genes (including *Il10*) (29, 52, 53). Therefore, we proposed that PlGF drives the M2-like polarization of dMφs *via* the STAT3-C/EBPβ signaling pathway. This hypothesis was also verified by the positive correlation between the expression level of C/EBPβ and the phosphorylation level of STAT3 at Tyr 705 in PlGF-polarized dMφs, in addition to the abrogated effect of a selective STAT3 inhibitor on the increased C/EBPβ expression induced by PlGF. Thus, our study shed light on a molecular pathway orchestrating the M1/M2 balance in the early decidua, which is initiated by PlGF and ends in the up-regulating expression of M2-associated markers due to amplified STAT3-C/EBPβ signaling. However, the factors regulating PlGF secretion by DSCs are still unknown. In the near future, we will try to explore the upstream mechanism involved in the expression and secretion of PlGF in DSCs.

**Figure 6:**
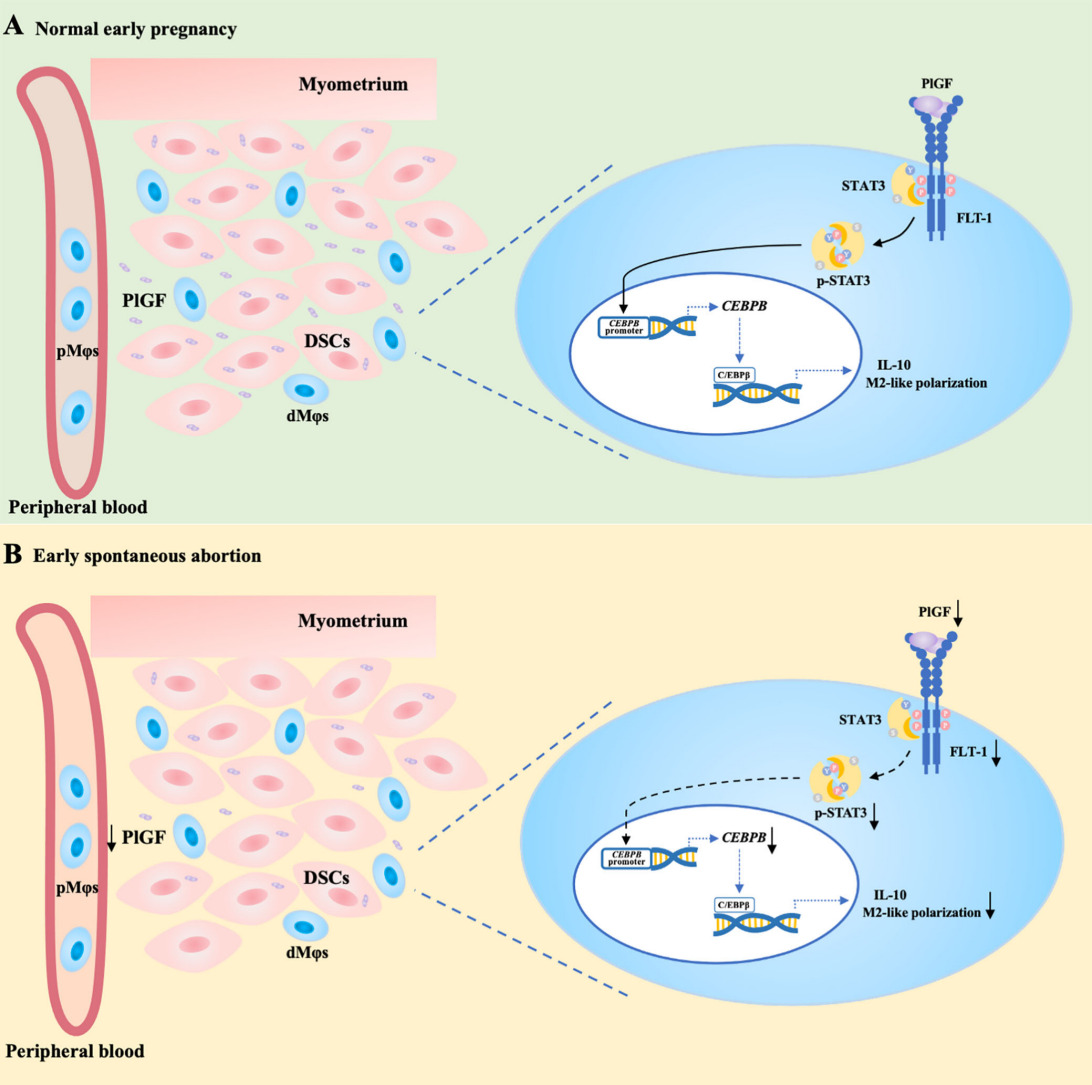
PlGF/FLT-1 deficiency leads to reduced STAT3-C/EBPβ signaling and aberrant polarization in decidual macrophages during early spontaneous abortion. **(A)** In normal pregnancy, PlGF secreted by decidual stromal cells (DSCs) promotes the M2-like polarization and IL-10 production in decidual macrophages (dMφs) by binding to its receptor FLT-1 expressing on dMφs and thereby up-regulating the level of p-STAT3, which induces *CEBPB* transcription. **(B)** In early spontaneous abortion, the deficiency of PlGF/FLT-1 leads to the decreased tyrosine phosphorylation and transcriptional activity of STAT3, then the reduced C/EBPβ expression, and eventually the aberrant polarization of dMφs. p-STAT3, phosphorylated STAT3.

In conclusion, acting as a link between DSCs and dMφs, PlGF stimulates its receptor FLT-1 on dMφs, activates the STAT3-C/EBPβ signaling pathway, and eventually drives the M2-like polarization of dMφs. PlGF is one of the crucial factors contributing to the development of maternal immune tolerance and the maintenance of early pregnancy. The decreased PlGF secretion by DSCs may result in spontaneous abortion in the first trimester. As shown in **Figure 6A**, we demonstrated that PlGF skews dMφs toward an M2-like phenotype and stimulates IL-10 production from dMφs *via* the FLT-1-STAT3-C/EBPβ axis. Remarkably, the deficiency of PlGF/FLT-1 in the early decidua is probably responsible for the decreased tyrosine phosphorylation and transcriptional activity of STAT3, the down-regulated expression of C/EBPβ, the polarization disturbance of dMφs, and ultimately the increased risk of early spontaneous abortion (**Figure 6B**). These findings expanded our understanding of the regulatory role of PlGF in maintaining early pregnancy and the underlying mechanism. Excitingly, our results highlight a rationale that PlGF is a promising target to prevent early spontaneous abortion. In future work, the regulatory role of PlGF in dMφs and early pregnancy should be further validated by the conditional knockout of macrophage *Flt1* in *Pgf* knockout mice.

## Data availability statement

The original contributions presented in the study are included in the article/**Supplementary Material**. Further inquiries can be directed to the corresponding authors.

## Ethics statement

The studies involving human participants were reviewed and approved by the Research Ethics Committee of the Second Affiliated Hospital, Chongqing Medical University. The patients/participants provided their written informed consent to participate in this study. The animal study was reviewed and approved by the Animal Care and Use Committee of Chongqing Medical University. Written informed consent was obtained from the individual(s) for the publication of any potentially identifiable images or data included in this article.

## Author contributions

Concept and design: LH, FH, and RC. Data collection: RC, JD, LW, and LJ. Construction of *in vivo* models: RC, HL, HJ and XL. Data analysis: RC and FH. Writing - original draft: RC. Writing - review and editing: FH. All authors contributed to the article and approved the submitted version.
