# Supplementary material for: PlGF/FLT-1 deficiency leads to reduced STAT3-C/EBPβ signaling and aberrant polarization in decidual macrophages during early spontaneous abortion
Source: Front Immunol. 2023 Mar 15;14:1061949. doi: 10.3389/fimmu.2023.1061949 (PMC10074254; doi:10.3389/fimmu.2023.1061949)
Supplement: Supplementary file 1 [file Table_1.docx]

**Supplemental Table 1 Demographic and clinical characteristics of study participants.**

|  | **Normal pregnancy**  (n=85) | **Spontaneous abortion**  (n=31) | ***P* Value** |
| --- | --- | --- | --- |
| **Sex,** N (%) Female | 85 (100%) | 31 (100%) | N/A |
| **Maternal age,** years  (IQR) | 28 (25-33) | 31 (26-33) | 0.264 |
| **Gestational age,** weeks  (IQR) | 7.14 (6.57-7.71) | 8.57 (7.43-9.43) | ＜0.001 |
| **Crown-rump length,** mm  (IQR) | 4.0 (3.0-5.0) | 3.4 (2.0-5.0) | 0.514 |
| **Gravidity,** median (IQR) | 2 (1-3) | 2 (1-3) | 0.670 |
| **Parity,** median (IQR) | 1 (0-1) | 0 (0-1) | 0.07 |
| **Miscarriage history, N** (%) | 18 (21.2%) | 15 (48.4%) | 0.04 |

Either the t-test or Wilcoxon Rank Sum test or Pearson’s chi-squared test was used where appropriate.

IQR, interquartile range.
