# Supplementary material for: PlGF/FLT-1 deficiency leads to reduced STAT3-C/EBPβ signaling and aberrant polarization in decidual macrophages during early spontaneous abortion
Source: Front Immunol. 2023 Mar 15;14:1061949. doi: 10.3389/fimmu.2023.1061949 (PMC10074254; doi:10.3389/fimmu.2023.1061949)
Supplement: Supplementary file 2 [file Table_2.docx]

**Supplemental Table 2 Primers used for qRT-PCR.**

| **Gene symbol** | **Species** | **Sense primer (5ʹ→3ʹ)** | **Antisense primer (5ʹ→3ʹ)** |
| --- | --- | --- | --- |
| *FLT1* | *H. sapiens* | TTTGCCTGAAATGGTGAGTAAGG | TGGTTTGCTTGAGCTGTGTTC |
| *Pgf* | *M. musculus* | GGATGTGCTCTGTGAATGCAG | AATACGTGAGCTGGGTGTGG |
| *CEBPB* | *H. sapiens* | GGCCGGTTTCGAAGTTGATG | GCTGACAGTTACACGTGGGT |
| *PPARGC1A* | *H. sapiens* | TCTGAGTCTGTATGGAGTGACAT | CCAAGTCGTTCACATCTAGTTCA |
| *PPARGC1B* | *H. sapiens* | GATGCCAGCGACTTTGACTC | ACCCACGTCATCTTCAGGGA |
| *IRF4* | *H. sapiens* | CGCTCCTCTGTTTGTTTGGC | GTGCAGTTCCGTAGTCAGCT |
| *IRF5* | *H. sapiens* | GGGCTTCAATGGGTCAACG | GCCTTCGGTGTATTTCCCTG |
| *STAT1* | *H. sapiens* | ATCAGGCTCAGTCGGGGAATA | TGGTCTCGTGTTCTCTGTTCT |
| *STAT6* | *H. sapiens* | GTTCCGCCACTTGCCAATG | TGGATCTCCCCTACTCGGTG |
| *GAPDH* | *H. sapiens* | ATCTCTGCCCCCTCTGCTG | CATCACGCCACAGTTTCCC |
| *GAPDH* | *M. musculus* | GTCAAGGCCGAGAATGGGAA | CTCGTGGTTCACACCCATCA |
