# Supplementary material for: PlGF/FLT-1 deficiency leads to reduced STAT3-C/EBPβ signaling and aberrant polarization in decidual macrophages during early spontaneous abortion
Source: Front Immunol. 2023 Mar 15;14:1061949. doi: 10.3389/fimmu.2023.1061949 (PMC10074254; doi:10.3389/fimmu.2023.1061949)
Supplement: Supplementary file 3 [file Table_3.docx]

**Supplemental Table 3 Fluorescent antibodies used for flow cytometry.**

| Fluorescent antibody | Clone | Supplier | Identifier | Type |
| --- | --- | --- | --- | --- |
| APC anti-human/mouse/rat Vimentin | 280618 | R&D Systems | Cat#IC2105A | Intracellular staining |
| BV510 anti-human CD45 | HI30 | BioLegend | RRID:AB_2561383 | Cell surface staining |
| BV421 anti-human CD45 | HI30 | BioLegend | RRID:AB_2561357 | Cell surface staining |
| Pacific Blue anti-mouse CD45 | 30-F11 | BioLegend | RRID:AB_493535 | Cell surface staining |
| FITC anti-human CD14 | HCD14 | BioLegend | RRID:AB_830677 | Cell surface staining |
| BV421 anti-human CD14 | HCD14 | BioLegend | RRID:AB_2563296 | Cell surface staining |
| PE/CY7 anti-human CD86 | BU63 | BioLegend | RRID:AB_2728392 | Cell surface staining |
| PE/CY7 anti-human CD206 | 15-2 | BioLegend | RRID:AB_10933248 | Cell surface staining |
| PE/CY7 anti-human IL-10 | JES3-9D7 | BioLegend | RRID:AB_2125385 | Intranuclear staining |
| APC anti-human TGF-β1 | TW4-2F8 | BioLegend | RRID:AB_10682896 | Intranuclear staining |
| APC anti-human CD80 | 2D10 | BioLegend | RRID:AB_2076147 | Cell surface staining |
| APC anti-human CD86 | BU63 | BioLegend | RRID:AB_2721449 | Cell surface staining |
| APC anti-human CD209 | 9E9A8 | BioLegend | RRID:AB_1134045 | Cell surface staining |
| Alexa Fluor 647 anti-human IL-1β | JK1B-1 | BioLegend | RRID:AB_604135 | Intranuclear staining |
| PE anti-human FLT-1 | 49560 | R&D Systems | RRID:AB_2247234 | Cell surface staining |
| FITC anti-mouse F4/80 | BM8 | BioLegend | RRID:AB_893502 | Cell surface staining |
| APC anti-mouse FLT-1 | 141522 | R&D Systems | RRID:AB_622149 | Cell surface staining |
| Alexa Fluor 488 anti-Stat3 | D3Z2G | CST | RRID:AB_2728821 | Intranuclear staining |
| Alexa Fluor 647 anti-Phospho-Stat3 (Tyr705) | D3A7 | CST | RRID:AB_10694637 | Intranuclear staining |
| Alexa Fluor 647 anti-Phospho-Stat3 (Ser727) | D4X3C | CST | RRID:AB_2799811 | Intranuclear staining |
| Alexa Fluor 647 anti-CEBP beta | E299 | Abcam | Cat#ab237415 | Intranuclear staining |

APC, allophycocyanin; BV510, brilliant violet 510; BV421, brilliant violet 421; FITC, fluorescein isothiocyanate; PE/CY7, phycoerythrin-canin7; TGF-β1, transforming growth factor-β1; PE, phycoerythrin; FLT-1, fms-like tyrosine kinase-1; Stat3, signal transducer and activator of transcription 3; CEBP beta, CCAAT enhancer-binding protein beta.
