# Supplementary material for: PlGF/FLT-1 deficiency leads to reduced STAT3-C/EBPβ signaling and aberrant polarization in decidual macrophages during early spontaneous abortion
Source: Front Immunol. 2023 Mar 15;14:1061949. doi: 10.3389/fimmu.2023.1061949 (PMC10074254; doi:10.3389/fimmu.2023.1061949)
Supplement: Supplementary file 4 [file Table_4.docx]

**Supplemental Table 4 Primers for the construction of overexpression vectors.**

| **Vector** | **Primer** | |
| --- | --- | --- |
| CMV enhancer-MCS-SV40-Puro-*STAT3* | Forward  Reverse | GTTTAAACGGGCCCTCTAGACGCCACCATGGCCCAATGGAATCAGCTAC  CAGCGGTTTAACTATCTAGATCACATGGGGGAGGTAGCGCACTC |
| MCS-firefly Luc-*CEBPB* | Forward  Reverse | TTTCTCTATCGATAGGTACCATGCATTCCTTCACTGGTAAATG  CTTAGATCGCAGATCTCGAGGACCCTCGGGTGGGTCCCCTTCC |
| MCS-firefly Luc-Mutant *CEBPB* | Forward  Reverse | TTTCTCTATCGATAGGTACCATGCATTCCTTCACTGGTAAATG  CTTAGATCGCAGATCTCGAGGACCCTCGGGTGGGTCCCCTTCC |
